# New Appliances and Things Medical

**Published:** 1896-04-25

**Authors:** 


### NEW APPLIANCES AND THINGS MEDICAL.

### MARVINO.

(Dowden and Co., St. Martin's Lane, London, E.C.)

Under the name of Marvino, Messrs. Dowden and Co. are introducing into England a pure grape wine of a generous Burgundy character, and, from an examination of samples of this wine, we have been able to testify to its excellent character. Our analytical figures are as follows: Ash 0·24 per cent., containing iron, and a trace of manganese, showing the Burgundy characteristics of the wine; alcohol 8·30 per cent. by weight. This quantity is somewhat lower than that which we have found in some recent Burgundies, but is still sufficiently high to show that the fermentation has been allowed to proceed for a normal period, as the accompanying sugar amounts to only 0·32 per cent. glucose. The total extract was found to amount to 2·37 per cent., and the acidities were similar to those of wines of this character. There has been a large increase within the last few years of these cheaper Burgundies and clarets into the English market, and members of the profession are much inclined to advise the use of them.

